# Risk Factors for Fatality among Confirmed Adult Dengue Inpatients in Singapore: A Matched Case-Control Study

**DOI:** 10.1371/journal.pone.0081060

**Published:** 2013-11-22

**Authors:** Tun-Linn Thein, Yee-Sin Leo, Dale A. Fisher, Jenny G. Low, Helen M. L. Oh, Victor C. Gan, Joshua G. X. Wong, David C. Lye

**Affiliations:** 1 Communicable Disease Center, Tan Tock Seng Hospital, Singapore, Singapore, Singapore; 2 Yong Loo Lin School of Medicine, National University of Singapore, Singapore, Singapore; 3 Saw Swee Hock School of Public Health, National University of Singapore, Singapore, Singapore; 4 Department of Medicine, National University Hospital, Singapore, Singapore; 5 Department of Infectious Diseases, Singapore General Hospital, Singapore, Singapore; 6 Department of Medicine, Changi General Hospital, Singapore, Singapore; D'or Institute of Research and Education, Brazil

## Abstract

**Objectives:**

To identify demographic, clinical and laboratory risk factors for death due to dengue fever in adult patients in Singapore.

**Methods:**

Multi-center retrospective study of hospitalized adult patients with confirmed dengue fever in Singapore between 1 January 2004 and 31 December 2008. Non-fatal controls were selected by matching age and year of infection with fatal cases. World Health Organization 1997, 2009 criteria were applied to define dengue hemorrhagic fever (DHF), warning signs and severe dengue. Statistical significance was assessed by conditional logistic regression modeling.

**Results:**

Significantly more fatal cases than matched controls had pre-existing co-morbid conditions, and presented with abdominal pain/tenderness. Median pulse rates were significantly higher while myalgia was significantly less frequent in cases. . Fatal cases also had higher leucocyte counts, platelet counts, serum sodium, potassium, urea, creatine and bilirubin levels on admission compared to controls. There was no statistical significant difference between the prevalence of DHF and hematocrit level among cases and controls. Multivariate analysis showed myalgia and leucocyte count at presentation were independent predictors of fatality (adjusted odds ratios 0.09 and 2.94 respectively). None of the controls was admitted to intensive care unit (ICU) or given blood transfusion, while 71.4% and 28.6% of fatal cases received ICU admission and blood transfusion.

**Conclusions:**

Absence of myalgia and leucocytosis on admission were independently associated with fatality in our matched case-control study. Fatalities were also commonly associated with co-morbidities and clinicians should be alarmed if dengue patients fulfilled severe dengue case definition on admission.

## Introduction

Dengue infection is caused by one of four dengue virus serotypes and transmitted by the *Aedes* mosquito. Over 2.5 billion people in tropical and subtropical regions worldwide are at risk of the disease. With no effective vaccine or antiviral treatment, close observation and judicious fluid therapy remain crucial [1]. The fatality rate due to dengue shock syndrome (DSS), the most severe form of dengue disease may be reduced to as low as <0.2% with careful management [2]. Understanding the risk factors for progression to severe dengue and death is essential in determining triage and management algorithms.

Risk factors for fatality in patients with dengue infection have been identified in studies in the Americas [3-8], Africa [9], Australia [10], Pacific islands [11], South Asia [12,13] and Southeast Asia [14-17]. In addition to endemic countries, fatal imported cases have been reported in Norway and Germany [18,19]. Significant risk factors associated with fatality reported were hematemesis and melena [3], tachycardia on admission [20], profound thrombocytopenia and leucocytosis [15]. Juneja et al reported that APACHE II may predict higher risk for death among dengue patients admitted to intensive care unit (ICU)[12]. In Cuba, dengue hemorrhagic fever (DHF) and DSS fatality and hospitalization rates were the highest among young infants and the elderly[6].

Singapore is hyperendemic for dengue , with all four serotypes circulating [21]. Deaths from dengue have required government notification since 1977 [20,22,23]. The case fatality rate of dengue in Singapore is among the lowest in the world. We previously reported the findings from our multi-center adult dengue death cohort study across the years 2004-2008 in Singapore in which dengue infection was confirmed by the presence of dengue virus RNA by polymerase chain reaction (PCR) or non-structural protein 1 (NS1) antigen in serum [24]. Our dengue death cohort revealed fatality occurred most commonly among older patients with co-morbidities.

This case-control study was intended to: (1) compare demographic, clinical and laboratory features of fatal and non-fatal adult dengue patients, (2) compare warning signs and dengue severity classifications among fatal and non-fatal dengue patients, and (3) identify predictors of dengue fatality on hospital admission.

## Methods

### Ethics statement and study population

The retrospective adult dengue death study was approved by the Domain Specific Review Board of National Healthcare Group (Tan Tock Seng Hospital, National University Hospital and Alexandra Hospital) as well as the Institutional Review Board of SingHealth Group (Singapore General Hospital and Changi General Hospital)[24]. For controls, we collated demographic, clinical, laboratory, treatment and outcome data from laboratory-confirmed adult dengue cases managed by the Department of Infectious Disease, Tan Tock Seng Hospital, Singapore from 1 January 2004 and 31 December 2008 (DSRB B/05/115 & DSRB E/08/567). All data were collected by approved waiver of informed consent. Laboratory-confirmation was by dengue PCR or NS1 antigen assays. To determine matching conditions for controls, initial univariate analysis was performed which showed age was significantly different among fatal cases and non-fatal controls (median 59 vs. 31 years, p<0.001). Controls were subsequently matched for age (+ 5 years) and year of admission to control for different predominant circulating dengue serotypes. We matched three non-fatal control patients for every fatal case; however, a 1:2 ratio was used for one matched set from 2006 and three matched sets from 2007 because of lack of suitable controls. However, this would not have effect on the power of statistical tests done [25].

### Definitions

Dengue hemorrhagic fever was defined according to the World Health Organization (WHO) 1997 guideline as having fever or history of fever, presence of bleeding, thrombocytopenia (platelet count less than or equal to 100x10^9^/L) and evidence of plasma leakage [26]. The subjects were classified as have warning signs according to the WHO 2009 guideline if they had abdominal pain or tenderness, persistent vomiting, clinical fluid accumulation, mucosal bleeding, lethargy, hepatomegaly and hematocrit rise concurrent with a rapid drop in platelet count. Severe dengue according to the WHO 2009 criteria comprised severe plasma leakage, severe bleeding or severe organ involvement [27]. Pitt bacteremia scores (PBS) [28] were determined for cases and controls as a measure of acute illness severity. Patients were classified as critically ill if they had a total of > 4 points [28].

### Statistical methods

Data were entered into a Microsoft Access 2003 software database (Microsoft Corp., Redmond, WA, USA). Data audit was done by a separate research assistant and any discrepancies were resolved by the investigators. The descriptive results for categorical variables were displayed by frequency and percentage. For continuous variables, median and ranges were used. Data analyses were done using SPSS software version 16 (SPSS Inc., Chicago, IL, USA) with the level of significance set at a two-tailed *p* value of <0.05 assessed by conditional logistic regression modeling [29]. Six readily available clinical parameters (symptoms, signs and full blood counts) with p<0.05 from univariate analysis were put into the full multivariate model. The final multivariate model with two variables was selected by testing for parsimony using the likelihood ratio test. There was no significant difference between our full and final models (p=0.17).

## Results

### Cohort description

There were 28 fatal confirmed cases between 2004 and 2008 (range 2-9/year, age range 21-86 years) and 80 age-matched non-fatal hospitalized control patients. Dengue was confirmed by PCR in all but one which was positive by NS1 antigen testing. Cases and controls had similar ethnic profiles. Fatal cases had medical co-morbidities more frequently (75% vs. 51.3%; p<0.05) particularly cardiac and renal disorders (Table 1).

**Table 1:** Characteristics of patients and clinical manifestations at the time of hospital presentation.

**Variables**	**Non-Fatal (n=80)**	**Fatal (n=28)**	Odds ratio (95% confidence interval)*	*p* value*
**Gender and race**				
Male gender	40 (50.0)	19 (67.9)		0.080
Chinese	62 (77.5)	19 (67.9)		0.302
**Co-morbidities**				
Diabetes mellitus	17 (21.2)	9 (39.1)		0.078
Hypertension	35 (43.8)	12 (50.0)		0.821
Heart failure	2 (2.5)	2 (11.1)		0.176
Hyperlipidemia	16 (20.0)	3 (16.7)		0.503
Cardiac disorder	7 (8.8)	10 (47.6)	10.643 (2.274 - 49.821 )	0.003
Renal disorder	2 (2.5)	8 (40.0)	21.176 ( 2.63-170.54)	0.004
**Clinical features**				
Days of illness at presentation	4 ( 1 - 8 )	4 ( 1 - 10 )		0.210
Fever symptom on admission	79 (98.8)	21 (75.0)	0.050 ( 0.006 - 0.405)	0.005
Chills/rigors	21 (33.9)	5 (17.9)		0.373
Nausea	34 (42.5)	7 (25.0)		0.110
Vomiting	32 (40.0)	7 (25.0)		0.174
Diarrhea	30 (37.5)	7 (25.0)		0.244
Myalgia	58 (72.5)	7 (25.0)	0.185 ( 0.076 - 0.449)	<0.001
Arthralgia	19 (23.8)	0 (0.0)		0.096
Hemorrhagic manifestation	14 (17.5)	5 (17.9)		>0.9
Systolic blood pressure (mmHg)	130.0 ( 87-200 )	139.5 ( 105.0-230.0 )		0.105
Pulse rate /minute	86.5 ( 60-135 )	102.0 ( 61.0-160.0 )	1.046 ( 1.018 - 1.075 )	0.001
Respiratory rate /minute	20 ( 15-25 )	20 ( 16.0-42.0 )		0.220
Oxygen saturation (%)	990 ( 95-100 ).	98.5 ( 80.0-100.0 )		0.136
**Laboratory investigations**				
Leucocyte count (10^9^/L)	2.5 ( 1.1 - 7.6 )	8.8 ( 2.8 - 31.7 )	2.07 ( 1.346 - 3.184 )	0.001
Hematocrit (%)	41.4 ( 28.6 - 53.5 )	41.2 ( 26.7 - 66.5 )		0.960
Platelet count (10^9^/L)	68 ( 23 - 229 )	91 ( 16 - 431 )	1.011 ( 1.003 - 1.02 )	0.006
Sodium (mmol/L)	134 ( 123 - 146 )	137 ( 119 - 150 )	1.138 ( 1.012 - 1.28 )	0.031
Potassium (mmol/L)	3.45 ( 2.7 - 5.4 )	4.35 ( 3.2 - 7.1 )	7.83 ( 2.514 - 24.386 )	<0.001
Urea (mmol/L)	4 ( 1.1 - 11 )	8.65 ( 4.2 - 28.3 )	1.705 ( 1.28 - 2.27 )	<0.001
Creatinine (umol/L)	86.5 ( 43 - 157 )	125.5 ( 71 - 1175 )	1.032 ( 1.013 - 1.051 )	0.001
Protein (g/L)	65 ( 50 - 78 )	62 ( 29 - 75 )		0.139
Albumin (g/L)	36 ( 27 - 46 )	33 ( 17 - 93 )		0.199
Bilirubin (umol/L)	11 ( 4 - 26 )	17 ( 4 - 607 )	1.077 ( 1.009 - 1.15 )	0.025
Aspartate aminotransferase (U/L)	96 ( 22 - 1388 )	90 ( 15 - 12600 )		0.135
Alanine aminotransferase (U/L)	63 ( 14 - 1024 )	50.5 ( 3 - 2254 )		0.112

Median (range) for continuous variables, n (%) for categorical variables

Cardiac disorder=ischaemia and other heart diseases excluding heart failure and peripheral vascular diseases: renal disorders=renal failure, calculi and other kidney diseases

* Conditional logistic regression modeling

### Clinical features on admission

The duration of illness preceding hospital admission was similar between cases and controls at a median of 4 days. Fatal cases were lesss often febrile (p<0.05) and reported myalgic less frequently (p<0.001). Fatal cases also had a higher median pulse rate (p<0.01) and higher median values of leucocyte and platelet counts, serum sodium, potassium, urea, creatinine and bilirubin (Table 1).

Cases had abdominal pain and tenderness more often than controls (p<0.05). More cases fulfilled the WHO 2009 severe dengue case definition than controls (p<0.01). Proportions of DHF did not differ significantly between cases and controls. More cases than controls had a Charlson score greater than or equal to 3 which predicts one year mortality [30] and had PBS of >4 (p<0.05) (Table 2).

**Table 2:** Severity assessment at presentation.

**Variables**		**Non-Fatal (n=80)**	**Fatal (n=28)**	**Odds ratio (95% confidence interval)**	p value*
WHO 2009 warning signs					
	Abdominal pain/tenderness	12 (15)	9 (32.1)	2.993 ( 1.014 - 8.84 )	0.047
	Persistent vomiting	0 (0)	0 (0)		nd
	Clinical fluid accumulation	1 (1.2)	2 (7.1)		0.143
	Mucosal bleeding	5 (6.2)	0 (0)		0.432
	Malaise/lethargy	26 (32.5)	11 (39.3)		0.521
	Hepatomegaly	0 (0)	2 (7.1)		0.478
	HCT rise with rapid platelet drop	3 (3.8)	1 (3.7)		>0.999
	Any warning sign	39 (48.8)	19 (67.9)		0.071
Disease classifications					
	Dengue hemorrhagic fever	6 (7.5)	5 (17.9)		0.143
	Dengue shock syndrome	0 (0)	2 (7.1)		0.478
	Severe dengue	8 (10)	22 (78.6)	53.53 (7.17 - 399.72)	<0.001
	Charlson score >3	3 (3.8)	8 (28.6)	4.77 (1.14 - 19.99)	0.033
	Pitt bacteremia score >4	1 (1.6)	7 (25)	16.22 (1.95 - 135.24)	<0.001

WHO=World Health Organization, HCT=hematocrit,

n (%) for categorical variables

* Conditional logistic regression modeling

During hospitalization, intravenous fluid and platelet transfusion was more frequent in cases than controls (p<0.05). None of the controls received blood transfusions or were admitted to an intensive care unit (ICU), whereas 64.3% of cases were given blood transfusion and 71.4% were admitted to ICU at a median of 3 days (range 1-9 days) from hospital admission. Cases had a significantly longer length of hospital stay than controls (p<0.01)(Table 3).

**Table 3:** Interventions during hospitalization.

**Variables**	**Non-Fatal (n=80)**	**Fatal (n=28)**	**p value**
Intravenous fluid	59 (73.8)	26 (92.9)	0.003
Platelet transfusion	22 (27.5)	18 (64.3)	0.002
Blood transfusion	0 (0.0)	9 (32.1)	na
ICU admission	0 (0.0)	20 (71.4)	na
LOS ICU	na	3 (1-32)	na
LOS hospitalization	6 (3-10)	8.5 (2-34)	0.005

ICU=intensive care unit, LOS=length of stay

Median (range) for continuous variables, n (%) for categorical variables

*Conditional logistic regression modeling

Six readily available clinical parameters that had p-values <0.05 in the univariate analysis (presence of fever, myalgia, pulse rate, presence of abdominal pain/tenderness, white cell and platelet counts) were included in the multivariate model. Myalgia and leucocyte count on admission were found to be independent predictors of fatality (Table S1). Likelihood ratio test was used to obtain the final multivariate model which included two variables; myalgia and leucocyte count on admission, which had adjusted odds ratios of 0.09 and 2.94 respectively (Table 4).

**Table 4:** Risk Factors for fatality in adult dengue inpatients.

**Variables**	**Adjusted Odds Ratio (95% confidence interval)**	*p* value*
Myalgia	0.087 (0.012 - 0.625)	0.015
White cell count	2.938 (1.264 - 6.829)	0.012

* Conditional logistic regression modeling

There were eight cases with leucocyte counts exceeding 10x10^9^/liter during hospitalization. We studied them in further detail to exclude other infectious causes. Notably, only one of them was found to have bacteremia (alpha hemolytic *Streptococcus* infection). All eight cases died within nine days of dengue diagnosis.

## Discussion and Conclusion

Our previous series of fatal cases reported shock as the commonest cause of death followed by organ impairment (71.4% with acute renal impairment, 57.1% with impaired consciousness and 53.6% with severe hepatitis) [24]. A case-control study from Singapore reported tachycardia on admission as independently associated with death [20]. Another case-control study from Brazil reported patients with hypotension and shock to be at greater risk of progression to death (p<0.05) [8]. A Puerto Rican case series suggested that presentation with shock should alert doctors of the risk of severe dengue [5]. In our study, fatal cases had a significantly higher pulse rate on admission to hospital compared with controls. High hematocrit was reported as an independent predictor of fatality in a study from Brazil [31]. This was not the case in our study as we found similar hematocrit levels between cases and controls.

We performed a multivariate analysis by including symptoms, signs and laboratory parameters on admission which were significant in univariate analysis. We found that a unit increase in leucocyte count led to a significantly higher risk of fatality (adjusted odds ratio 2.94). Leucocytosis was identified as a warning sign of severe dengue in a study from Taiwan [15].Studies from India and Taiwan reported that this phenomenon may be associated with secondary bacterial infection [15,32]. However, only 4 (14.3%) of fatal cases had documented bacteremia in our study. Myalgia is known to be associated with dengue fever, however, its significance in disease severity is not well established. Lee et al from Taiwan reported in 2012 that although fatal cases had myalgia more frequently, statistical significance was not reached[15]. A study on 31 adult dengue patients with muscle involvement showed muscle dysfunction in dengue is benign, transient and self-limiting [33]. In our study, significantly fewer fatal cases had muscle pain, thus its presence is unlikely to be a risk factor for fatality. Less seriously ill patients who complained of myalgia might have been admitted to the hospital and contributed to lower odds towards fatality. This highlights the importance of objective clinical examinations and laboratory investigations in assessing and predicting disease severity.

Interestingly, patients who fulfilled criteria for critical illness with a PBS >4 points on hospital admission were found to be significantly associated with fatality in univariate analysis in our cohort. This score based on mental status, vital signs, requirement for mechanical ventilation, and recent cardiac arrest was initially derived to assess the severity of acute illness at the time of positive blood culture [28]. The score has shown to be useful in predicting fatality in septic shock. Its correlation with APACHE II score has been established [34] which was shown to predict death in dengue cases admitted to ICU in a study from India [12].

A study from Mexico using national surveillance data reported risk factors associated with fatality were hematemesis (relative risk [RR] 2.6) and melena (RR 2.2) [3]. Another study from Brazil reported gastrointestinal bleeding was associated with higher risk of death [8]. In our study, fatal cases had similar hemorrhagic manifestations as controls.

Our study on adult fatal dengue identified by multi-organ impairment as an important cause [24]. This is consistent with a report by Simmons et al [1]. However in our current case-control study, aspartate transaminase (AST) and alanine transaminase (ALT) were not useful in predicting fatal outcomes. This reflects the inability of AST and ALT to discriminate DHF or severe dengue cases reported in a retrospective study from Singapore [35]. Serum bilirubin levels of fatal cases were significantly higher in cases highlighting a need for further research on liver involvement in dengue fatality. Fatal cases had elevated levels of serum urea and creatinine at presentation similar to the reports from Taiwan but they were not independently associated with fatality [36,37].

In our study, severe dengue as defined in WHO 2009 criteria at hospital presentation was found to be associated with eventual fatality. Although cases had a higher proportion of DHF and DSS at presentation, it could not differentiate progression to death from controls. This supported previous reports on the lack of sensitivity of the WHO 1997 dengue severity criteria in adequately differentiating clinically severe from non-severe cases [5,20]. Interestingly, we observed a higher proportion of patients with warning signs at presentation in cases versus controls, but the presence of warning signs was not discriminatory.

One of the strengths of our study was that all dengue infections were confirmed by PCR or NS1. In contrast, previous reports on fatal dengue often relied on serology only. Most of these studies did not focus on fatality among adults or were limited to specific time periods. Our data came from five major public hospitals in Singapore across the years 2004 to 2008. In the years 2004-2008, 42,653 dengue cases were reported from Singapore [38]. In our cohort, the highest numbers of fatalities were nine and eight in the year 2005 and 2007 respectively, when Singapore had large dengue outbreaks. The predominant strains were serotype 1 in 2005 and serotype 2 in 2007 [39]. Our study lacks data on primary or secondary infection status. There was no serotype data for individual patients. None of the cases had autopsies performed. Finally, this study does not attempt to compare dengue management protocols at the different hospitals. Our relatively small number of fatal cases also limited the implementation of multivariate modelling to include more covariates that are statistically significant in the univariate analysis. However, our findings based on readily available clinical parameters could be useful in guiding clinicians in dengue case management.

In conclusion, the case fatality rate of dengue in Singapore is low. Fatal cases were significantly associated with existence of medical co-morbidities. Presentation with severe dengue as defined by the 2009 WHO guideline showed association with fatality, but not presentation with DHF/DSS. Absence of myalgia and leucocytosis at presentation to hospital were independently associated with fatality in our study.

## Supporting Information

Table S1 **Risk Factors for fatality in adult dengue inpatients full model.**
(DOC)
