# Supplementary material for: Risk Factors for Fatality among Confirmed Adult Dengue Inpatients in Singapore: A Matched Case-Control Study
Source: PLoS One. 2013 Nov 22;8(11):e81060. doi: 10.1371/journal.pone.0081060 (PMC3838336; doi:10.1371/journal.pone.0081060)
Supplement: Table S1 — Risk Factors for fatality in adult dengue inpatients full model. (DOC) [file pone.0081060.s001.doc]

Table S1. Risk Factors for fatality in adult dengue inpatients full model

| **Variables** | **Adjusted Odds Ratio (95% confidence interval)** | ***p* value*** |
| --- | --- | --- |
| Fever | 0.159 (0.000 - 93.135) | 0.571 |
| Myalgia | 0.047 (0.003 - 0.661) | 0.023 |
| Pulse rate | 1.026 (0.943 - 1.116) | 0.549 |
| Abdominal pain/tenderness | 0.571 (0.045 - 7.289) | 0.666 |
| White cell count | 3.263 (1.292 - 8.243) | 0.012 |
| Platelet count | 0.986 (0.959 - 1.014) | 0.327 |

Readily available clinical parameters with p value <0.05 from univariate analysis were included in the model

* Conditional logistic regression modeling
